# Protective Effects of Melon Extracts on Bone Strength, Mineralization, and Metabolism in Rats with Ovariectomy-Induced Osteoporosis

**DOI:** 10.3390/antiox8080306

**Published:** 2019-08-14

**Authors:** Bongju Kim, Sung-Ho Lee, Su-Jin Song, Won Hyeon Kim, Eun-Sung Song, Jae-Chang Lee, Sung-Jae Lee, Dong-Wook Han, Jong-Ho Lee

**Affiliations:** 1 Dental Life Science Research Institute & Clinical Translational Research Center for Dental Science, Seoul National University Dental Hospital, Seoul 03080, Korea; 2 Department of Oral and Maxillofacial Surgery, School of Dentistry, Seoul National University, Seoul 03080, Korea; 3 Department of Cogno-Mechatronics Engineering, College of Nanoscience & Nanotechnology, Pusan National University, Busan 46241, Korea; 4 Bio-based Chemistry Research Center, Korea Research Institute of Chemical Technology, Ulsan 44429, Korea; 5 Department of Biomedical Engineering, Inje University, Gimhae 50834, Korea

**Keywords:** melon extract, antioxidant, osteoporosis, bone strength, bone mineralization, bone metabolism

## Abstract

A polyphenolic extract from melon (*Cucumis melo* L.), as a potential source of natural antioxidants, has been reported to have a positive effect on osteoblast activity. In this study, the protective effects of heat-treated melon extract (ECO-A) on bone strength, mineralization, and metabolism were examined in osteoporotic rat models. Osteoporosis was induced by ovariectomy (OVX) in female rats and then maintained for 8 weeks, along with the ingestion of phosphate-buffered saline (PBS, OVXP) or ECO-A (OVXE) for an additional 4 weeks. At a pre-determined timepoint, bone strengths, as well as bone mineral contents (BMC) and the density (BMD) of femurs and/or lumbar spines extracted from each animal, were measured by a mechanical test and dual-energy X-ray absorptiometry, respectively. Moreover, several biochemical markers for bone turnover were analyzed by respective colorimetric assay kits in addition to clinical analyses. The maximum load and stiffness of femurs from the OVXE group were found to be significantly higher than the other groups. Furthermore, the OVXE group showed significantly higher BMC, BMD, and bone volume than the OVX and OVXP groups, which were comparable to the non-OVX (sham) group. The levels of bone formation and resorption markers in the OVXE group were similar to the sham group, but significantly different from other groups. In conclusion, these results suggest that ECO-A can play potentially positive roles in the protection of bone loss in rats with OVX-induced osteoporosis.

## 1. Introduction

Osteoporosis or bone loss is the most common of all metabolic bone disorders in humans, representing a major public health problem [1,2]. The World Health Organization (WHO) has defined osteoporosis as a systemic skeletal disease characterized by a low bone mass and the microarchitectural deterioration of bone tissue, with a consequent increase in bone fragility and susceptibility to fracture [1,3]. Additionally, the National Institutes of Health (NIH) defines it as a skeletal system disease that increases the risk of fracture due to the weakening of bone strength [2,4]. Osteoporosis is classified into primary and secondary osteoporosis. Primary osteoporosis is classified into menopausal osteoporosis (type 1) and senile osteoporosis (type 2) for convenience, but it is difficult to classify correctly because the two types merge at a similar period [5]. While the declines in estrogen, generally following menopause, are well-known risk factors for osteoporosis in women, the effects of age-related testosterone decline in men on bone health are less well-known [6]. Although osteoporosis can be treated with medication, side effects such as gastrointestinal disorders, esophagitis, myalgia, jawbone necrosis, or atypical fracture have been reported [7,8]. Particularly, the use of testosterone therapy has been recommended in men with symptomatic low testosterone who are at a high risk of fracture [9], though this should be done in combination with medication with a proven antifracture effect, such as a bisphosphonate. As side effects have been reported for therapeutic agents used in osteoporosis patients, some alternative methods for the prevention of osteoporosis have been actively studied using various extracts or compounds available from natural sources [10]. Among these many natural substances with osteoporosis-relieving effects, mixed formulations of red clover and pomegranate [11] and *Artemisia annua* extract [12] have been shown to prevent ovariectomy (OVX)-induced osteoporotic bone loss. In addition, a recent report showed that curcumin could partially reverse changes in the diversity of gut microbiota caused by OVX-induced estrogen deficiency [13]. Furthermore, melon is reported to increase alkaline dephosphorylase activity in an aqueous extract and increase the expression of Runx2 and osterix, which are osteogenic markers [14]. Melon (*Cucumis melo* L.) is one member of the Cucurbitaceae family and an important diploid horticultural crop, and is cultivated worldwide in subtropical and tropical regions [15,16]. Melon is used as a tonic, laxative, galactagogue, diuretic, and diaphoretic, and its extract is known to exhibit high antioxidant activity, like superoxide dismutase [17,18]. Recent studies reported that the richest phytochemical was caffeic acid and that gallic acid, ellagic acid, catechin, ferulic acid, kaempferol, and chlorogenic acid were also the main phenolic compounds found in the extracts of melon peels and seeds [19,20]. Although water-soluble extracts of melon have positive effects on osteoblast differentiation [14], seminal evidence for their effects on osteoporosis is still inadequate.

In this study, we aimed to investigate the protective effects of heat-treated melon extract (ECO-A) on bone strength, mineralization, and metabolism in OVX-induced osteoporotic rats. For this purpose, the maximum load and stiffness of the femoral heads of the rats were measured by a mechanical strength test. Additionally, the bone mineralization of the whole body, femurs, and lumbar spines extracted from the animals fed with or without ECO-A was evaluated by measuring the bone mineral contents (BMC), density (BMD), and volume (BV) using dual-energy X-ray absorptiometry (DXA). Furthermore, we analyzed changes in the serum levels of C-telopeptide cross-linked collagen type I (CTXI) as a bone resorption marker, as well as alkaline phosphatase (ALP), osteocalcin (OC), and calcium as bone formation markers in the blood collected from each animal.

## 2. Materials and Methods

### 2.1. Melon Extract and Animals

ECO-A, prepared by extracting the whole melon including peels, seeds, and pulps with a heat treatment manufacturing process at 95–100 °C and 1 atm, was obtained from GL Biotech Co., Ltd. (Daejeon, South Korea). Twenty female adult (6-week-old) specific-pathogen-free Sprague Dawley (SD) rats (220–270 g in weight, Orient Bio, Gyeonggi-do, South Korea) were individually housed in metabolic cages during the adaptation period of 1 week before surgical treatment and until the day of sacrifice. They were fed with food (normal rodents pellet diet, Purinafeed, Seongnam-si, South Korea, Table S1) and water ad libitum both preoperatively and postoperatively under the conditions of a 12 h of day and night cycle, 21–23 °C temperature, and 40–60% humidity. All animal experiments related to surgical procedures and treatments were approved by the Animal Experiment Ethics Committee of Seoul National University and performed in accordance with the Animal Care and Use Committee guidelines (IACUC number: SNU-170706-4).

### 2.2. Surgical Procedures for Ovariectomy

A sterile surgical technique was applied throughout the study. Rats were anesthetized by intraperitoneal injection with chloropent (0.1 mL/kg) before OVX. After anesthetic induction, the lower abdomen was shaved and swabbed with alcohol and povidone iodine solutions. A longitudinal incision (2 cm long) was made on each of the ovaries, leading to exposition of the skin and muscle, and the fallopian tube was closed using 4-0 black silk suture (Silkam^®^, B. Braun Surgical S.A., Barcelona, Spain). Then, the fascia, muscle, and skin were sutured along the middle line incision using leftover suture, and then maintained for 8 weeks to induce osteoporosis.

### 2.3. Experimental Groups

**Figure 1:**
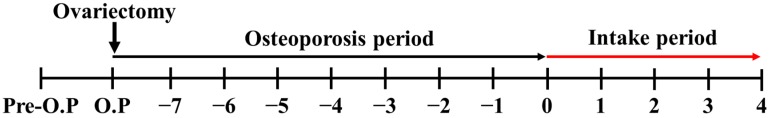
Experiment timeline (O.P: operation, Pre-O.P: pre-operation, 1 week before operation). After performing an ovariecomy in female Sprague Dawley (SD) rats, they were given regular food and water for 8 weeks and osteoporosis was induced. Then, phosphate-buffered saline (PBS) or heat-treated melon extract (ECO-A) was orally administered 3 times a day during the intake period.

Animals were randomly divided into four experimental groups: (1) sham group: those who ingested normal drinking water without any treatment during the entire experimental period, as the negative control; (2) OVX group: those who ingested normal drinking water for an additional 4 weeks after OVX on both ovaries; (3) OVXP group: those who ingested 1 x phosphate-buffered saline (PBS) for 4 additional weeks after OVX (OVX-PBS); and (4) OVXE group: those who ingested ECO-A for an additional 4 weeks after OVX (OVX-ECO-A). Experimental groups were perorally administered with 1 mL of either PBS or ECO-A using an oral gavage needle every morning (08:00), lunch (13:00), and evening (18:00) for 4 weeks (Figure 1). Body weight was weighed 3 days before surgery, on the day of surgery, and then every week thereafter. Water and food intake were measured daily for 5 weeks from the 7th week after OVX to the end of the experiment.

### 2.4. Mechanical Test

To compare the mechanical properties among experimental groups, femurs were extracted from each group (i.e., sham, OVX, OVXP, and OVXE groups (*n* = 5, respectively)). All tissue parts, such as muscles, ligaments, and cartilage, were removed from femur specimens and stored frozen at −10 °C using the gauze impregnated with physiological saline. Ten hours before the mechanical test, they were taken out and thawed at room temperature. In order to apply the vertical load to the femoral head, the distal part of the femur was fixed in the vertical direction using a dental resin (Vertex Trayplast, Vertex-Dental B.V., Soesterberg, Netherlands) and fully cured for 3 h. A metal jig with fixed specimens was attached to a hydraulic universal testing machine (MTS 858 Mini Bionix materials testing machine, MTS Systems Corp., Eden Prairie, MN, USA) with a load cell of a maximum load of 500 N (MTS 661.11B-02 Force Transducer, MTS Systems Corp.). Using a load jig with a thickness of 3 mm on the femoral head, the load was applied until the fracture of the femur occurred at a constant rate of 0.1 mm/min. The load–displacement curves were obtained in real time through mechanical tests. The test was terminated when the load suddenly dropped in the load–displacement curve, or cracks or fractures occurred in the specimen. The load at the breaking point was determined as the maximum load (N), and stiffness (N/mm) was measured from the change in displacement versus load variation in the elastic region.

### 2.5. Dual-Energy X-ray Absorptiometry

The regions of interest (ROI) of the left proximal femurs and lumbar spines 4–6 of each animal (*n* = 5, respectively) were set 2 days before sacrifice, as described elsewhere [21], and scanned using dual-energy X-ray absorptiometry (DXA) (InAlyzer, MEDIKORS Inc., Seoul, Korea). BMC (bone mass, g), BMD (BMC / unit area, g/cm^2^), and BV (cm^3^) were measured using the InAlyzer software (MEDIKORS Inc., Seongnam, Korea).

### 2.6. Blood Analysis

On the day of sacrifice, 2 mL blood from each group was collected by heart sampling using a sterile syringe (26 G, 3 mL), and plasma was separated after storage at room temperature for 1 h in vacuum tubes (BD Vacutainer^®^ SST™ II Advance, Franklin Lakes, NJ, USA). Serum was separated by centrifugation at 3000 rpm for 15 min and then stored in a deep freezer (−80 °C) prior to analysis. The level of C-telopeptide cross-linked collagen type I (CTXI) as a bone resorption marker was analyzed using a rat CTXI ELISA kit (Elabscience Biotechnology Inc., Houston, TX, USA). To analyze the levels of bone formation markers such as alkaline phosphatase (ALP), osteocalcin (OC), and calcium, a rat Alkaline Phosphatase Liver/Bone/Kidney (ALPL) ELISA kit (Elabscience Biotechnology Inc.), a rat Ostocalcin/Bone Gla Protein (OC/BGP) ELISA kit (Elabscience Biotechnology Inc.), and a calcium colorimetric assay kit (BioVision, Milpitas, CA, USA) were used, respectively, according to the manufacturer’s protocol.

### 2.7. Statistical Analysis

All quantitative data were expressed as means ± standard deviation. Statistical comparisons were carried out with a one-way analysis of variance (ANOVA) using the StatView ver. 5.0 software (Abacus Concepts Inc., Berkeley, CA, USA), which was followed by the Student–Newman–Keuls test for multiple comparisons. Statistical significance was accepted for a value of *p* < 0.05.

## 3. Results

### 3.1. Measurement of Body Weight and Daily Food and Water Intake

**Figure 2:**
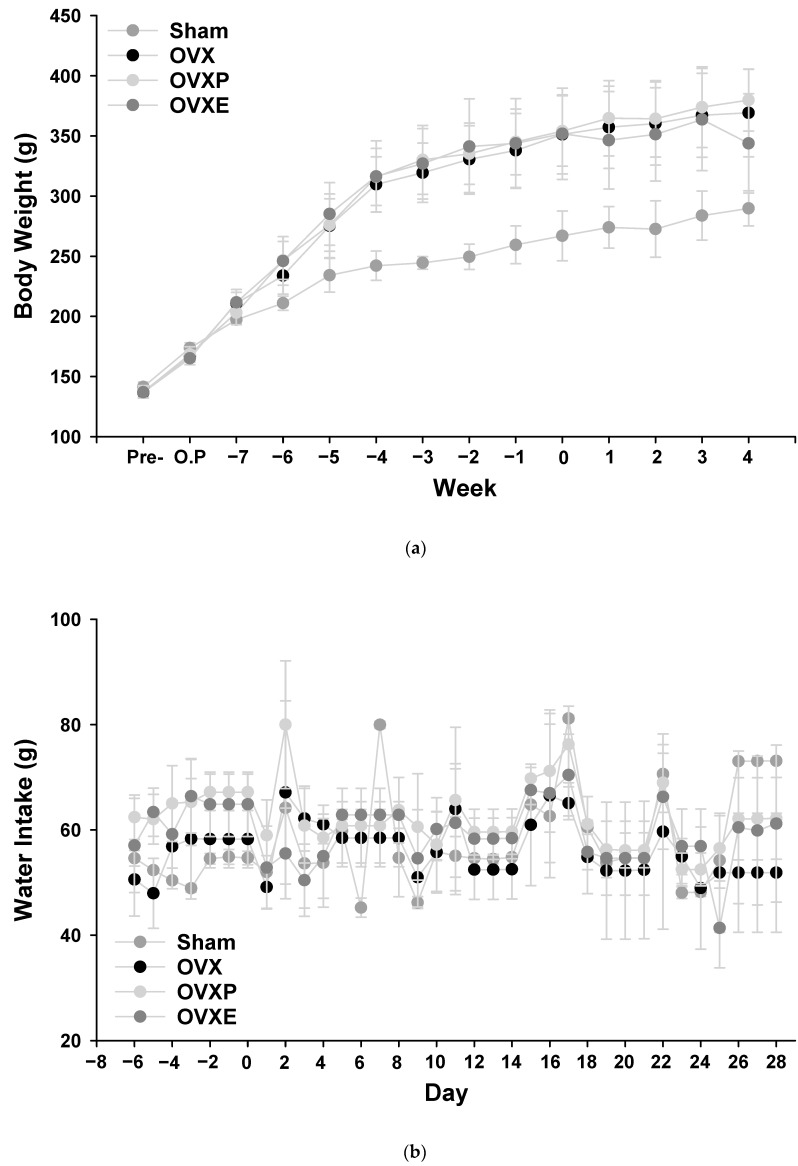

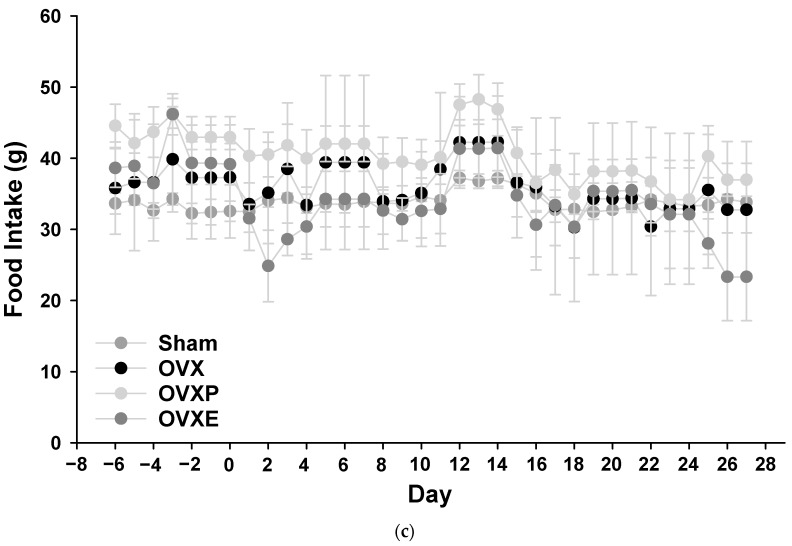
Changes in (**a**) body weight, (**b**) water intake, and (**c**) food intake. There was a significant difference in (**a**) between the sham and the other groups (*p* < 0.05) after 4 weeks, but not in (**b**) or (**c**).

After performing OVX on statistically similar rats before surgical procedures, an apparent difference in body weight between the non-OVX (sham) group and the other groups (OVX, OVXP, and OVE) began to appear from week 2. It was revealed that after 4 weeks of OVX, there was a significant difference in weight between the sham and other groups (*p* < 0.05), while no difference was observed among those groups (Figure 2a). In the case of food and water intake, there was no significant, and if any, negligible, difference between each group (Figure 2b,c).

### 3.2. Evaluation of Bone Strength

**Figure 3:**
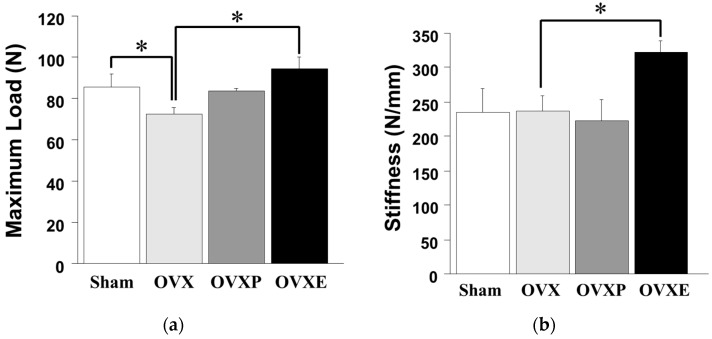
Strength testing results for the femoral head. (**a**) Maximum load and (**b**) stiffness. * *p* < 0.05.

Compared to the maximum load (85.68 ± 11.87 N) of femoral heads from the sham group, it was significantly (*p* < 0.05) decreased to 72.70 ± 0.02 N in OVX groups, which was calculated as a reduction of about 15%. The OVXP group also showed a significantly (*p* < 0.05) higher maximum load (83.64 ± 2.48 N) compared to the OVX group. Furthermore, the maximum load in the OVXE group was 94.33 ± 10.77 N, which was approximately 30% higher compared with that in the OVX group (Figure 3a). On the contrary, the stiffness values in the sham, OVX, and OVXP groups were 235.55 ± 67.68 N/mm, 236.48 ± 44.00 N/mm, and 223.20 ± 59.35 N/mm, respectively, showing that there was no significant difference. However, the OVXE group exhibited significantly (*p* < 0.05) greater stiffness (313.24 ± 37.71 N/mm) than the other groups, and the value was higher by more than 32% and 40% compared with the OVX and OVXP groups, respectively (Figure 3b).

### 3.3. Bone Mineralization

**Figure 4:**
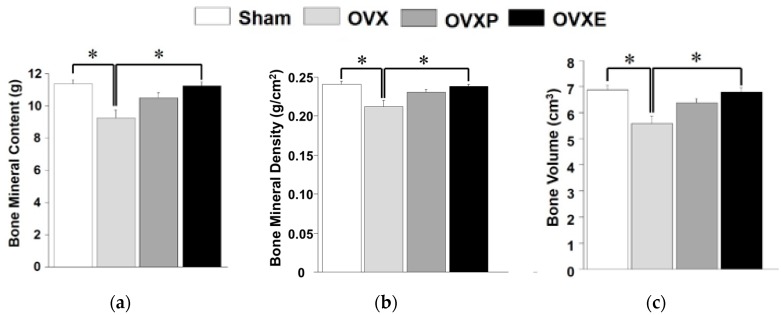
Analysis of dual-energy X-ray absorption for the whole body. (**a**) Bone mineral contents (BMC), (**b**) bone mineral density (BMD), and (**c**) bone volume (BV). * *p* < 0.05.

To compare the bone density among experimental groups, the ROI was scanned for the whole body, left femur, and lumbar vertebrae 4–6 by DXA. In the case of the whole body, compared to the BMC (11.36 ± 0.81 g) and BV (6.87 ± 0.49 cm^3^) of the sham group, BMC and BV were significantly (*p* < 0.05) decreased to 9.28 ± 1.45 g and 5.87 ± 0.80 cm^3^, respectively, in the OVX groups, corresponding to a reduction of more than 18% and 14%, respectively (Figure 4). However, the OVXP group showed 10.54 ± 0.73 g of BMC and 6.36 ± 0.47 cm^3^ of BV, which were significantly (*p* < 0.05) greater than the OVX group. Moreover, the BMC and BV of the OVXE group were 11.40 ± 0.59 g and 6.90 ± 0.13 cm^3^, respectively, which was approximately 22% higher compared with that of the OVX group. BMD analysis showed a very similar tendency with BMC and BV results. It was found that BMD results increased in the order of sham (0.24 ± 0.01 g/cm^2^), OVXE (0.24 ± 0.01 g/cm^2^), OVXP (0.23 ± 0.01 g/cm^2^), and OVX groups (0.21 ± 0.02 g/cm^2^).

**Figure 5:**
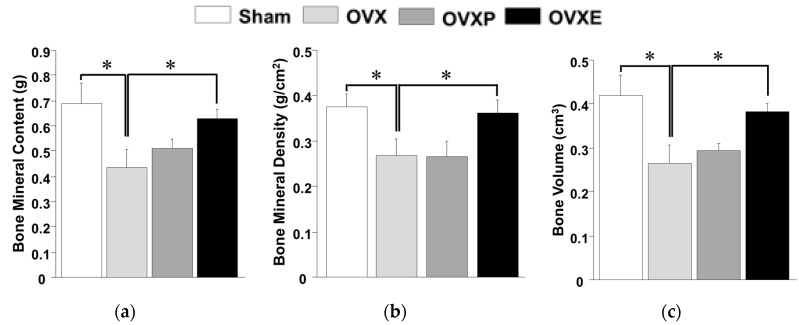
Analysis of dual-energy X-ray absorption for the left femur. (**a**) Bone mineral contents (BMC), (**b**) bone mineral density (BMD), and (**c**) bone volume (BV). * *p* < 0.05.

In the left femur, the results of BMC, BMD, and BV showed fairly similar patterns to those in the whole body (Figure 5). BMC (0.69 ± 0.08 g), BMD (0.38 ± 0.03 g/cm^2^), and BV (0.42 ± 0.05 cm^3^) in the sham group were significantly (*p* < 0.05) decreased to 0.44 ± 0.07 g, 0.27 ± 0.04 g/cm^2^, and 0.27 ± 0.04 cm^3^, respectively, in the OVX groups, whereas those values were considerably maintained in the OVXE groups (with 0.63 ± 0.04 g of BMC, 0.36 ± 0.03 g/cm^2^ of BMD, and 0.38 ± 0.02 cm^3^ of BV). On the other hand, BMC (0.51 ± 0.04 g), BMD (0.27 ± 0.03 g/cm^2^), and BV (0.29 ± 0.02 cm^3^) of the OVXP group were not significantly different from the OVX group.

**Figure 6:**
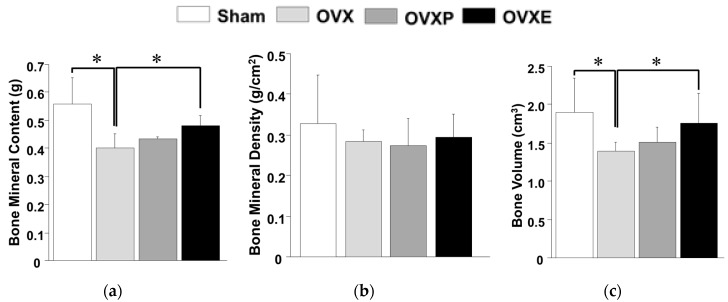
Analysis of dual-energy X-ray absorption for the lumbar vertebrae 4–6. (**a**) Bone mineral contents (BMC), (**b**) bone mineral density (BMD), and (**c**) bone volume (BV). * *p* < 0.05.

Interestingly, analyses for the lumbar vertebrae 4–6 were found to have considerably similar trends to the whole body and femur (Figure 6). Compared with BMC (0.56 ± 0.10 g), BMD (0.33 ± 0.12 g/cm^2^), and BV (1.90± 0.45 cm^3^) of the sham group, the values were significantly (*p* < 0.05) decreased to 0.40 ± 0.05 g, 0.28 ± 0.03 g/cm^2^, and 1.40 ± 0.12 cm^3^, respectively, in the OVX groups, which showed reductions of about 25% for BMC, 15% for BMD, and 18% for BV. In contrast, the values in the OVXE group were all noticeably maintained at 0.48 ± 0.04 g for BMC, 0.30 ± 0.06 g/cm^2^ for BMD, and 1.70 ± 0.40 cm^3^ for BV, which were all comparable to those in the sham group. There were no significant differences in the BMC, BMD, and BV levels between OVXP and OVX groups.

### 3.4. Blood Analysis

**Figure 7:**
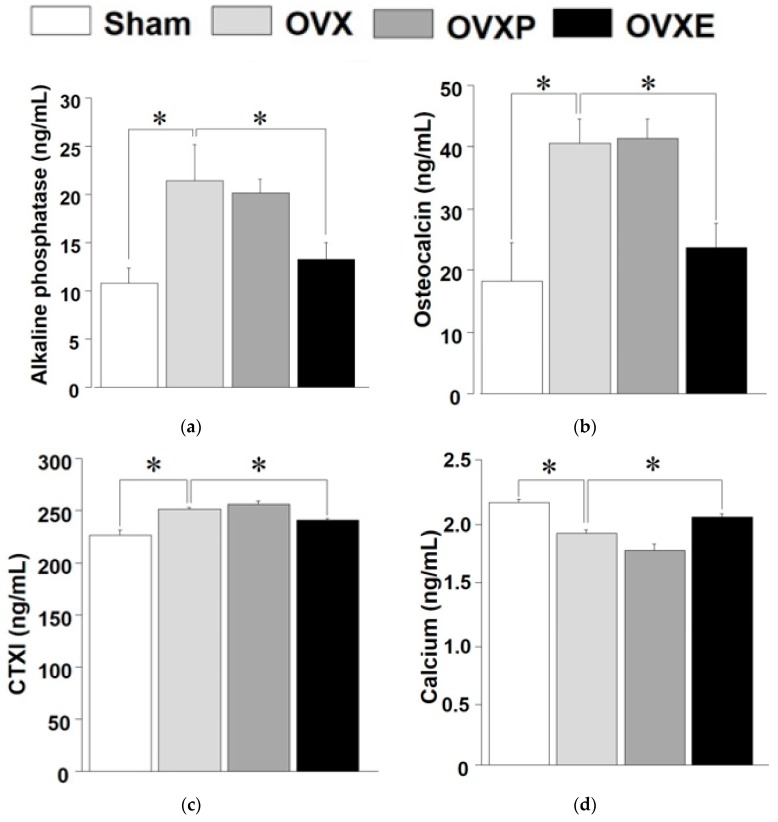
ELISA analysis of bone markers. (**a**) Alkaline phosphatase (ALP), (**b**) osteocalcin, (**c**) C-telopeptide cross-linked collagen type I (CTXI), and (**d**) calcium. * *p* < 0.05.

Compared with the contents of ALP (11.77 ± 2.88 ng/mL) and OC (18.33 ± 8.20 ng/m) in serum from the sham group, the contents were significantly (*p* < 0.05) increased to 21.15 ± 4.85 ng/mL and 40.72 ± 3.67 ng/mL, respectively, in the OVX groups, corresponding to an increase of over 60% in both (Figure 7a,b). The OVXP group showed almost equal results (20.99 ± 2.18 ng/mL of ALP and 41.45 ± 2.58 ng/mL of OC) to the OVX group, whereas those values were significantly (*p* < 0.05) decreased to 13.21 ± 3.18 ng/mL for ALP and 24.76 ± 2.80 ng/mL for OC in the OVXE group. Additionally, the content of CTXI (236.2 ± 9.02 ng/mL) in the sham group was significantly (*p* < 0.05) increased to 249.0 ± 5.87 ng/mL in the OVX group and 256.1 ± 7.90 ng/mL in the OVXP group. However, the OVXE group showed 240.2 ± 5.70 ng/mL of CTXI, which was significantly (*p* < 0.05) lower than the OVX and OVXP groups. (Figure 7c). On the contrary, the calcium content in the sham group was 2.09 ± 0.14 ng/mL, which was significantly (*p* < 0.05) decreased to 1.92 ± 0.12 ng/mL in the OVX group and 1.83 ± 0.22 ng/mL in the OVXP group, constituting a reduction of about 8% and 20%, respectively. In the OVXE group, the content was 2.01 ± 0.11 ng/mL, indicating a recovery to a level comparable to that of the sham group (Figure 7d).

## 4. Discussion

This study concentrates on examining the effect of ECO-A on bone metabolism in OVX-induced osteoporotic rat models. OVX in rats is a representative method of inducing menopausal disorders that reduce serum estrogen levels, and it is widely used in studies of osteoporosis and cardiovascular disease in women [22]. An artificial ovarian resection results in estrogen, a female hormone, becoming deficient and causing osteoporosis [23,24,25,26]. After OVX was performed in adult SD rats, the change in body weight was observed weekly. An apparent difference in weight between OVX and sham groups began to be observed from 2 weeks after surgery (Figure 2a). There are many reports stating that a decrease in estrogen secretion leads to an increase in weight [27,28,29], and this brings an increase in dietary intake, body fat accumulation, and consequently body weight [30,31,32]. It is suggested that ECO-A did not significantly affect weight loss, because no change in weight was observed in the OVXE groups administered with ECO-A after inducing osteoporosis.

Unlike other connective tissues, the mechanical strengths of bone are the result of the deposition of calcium phosphate in organic components. This highly specialized connective tissue is a strong support of the human body and plays an important role in the metabolic interrelationship. The strength of bone is the maximal amount of load tolerated before structural failure occurs. If bone strength is impaired, it is directly related to critical problems, including decreases in the amount of bone mass, as well as changes in the bone micro-architecture and in the biophysical properties of the bone tissue [33]. Therefore, to determine bone strength accurately, bone quality should be analyzed with a consideration of the micro-structural properties, the composition of mineral and collagen of bone, and microdamage [34]. In this study, the strength of femoral heads in the OVXE group was shown to be significantly higher compared with the OVX group, which implicates that ECO-A favorably affects bone strength (Figure 3). Nevertheless, there are several practical drawbacks that represent limitations. As the test specimens are small, it can be time-consuming to fix the distal part of the specimen to the vertical direction and align the specimen appropriately in the jig during the load application. Furthermore, a 500 N load cell was used since this is the equipment we have available, but a smaller load cell, in the 200–300 N range, would be more appropriate for future tests, allowing for a greater resolution and accuracy in the testing.

Previous studies have reported meaningful protective effects of natural extracts on osteoporosis after 4~12 weeks [10,11,35]. In this study, the potential effect of ECO-A was observed 4 weeks after OVX-induced osteoporosis, which is considered as a relatively early phase. Osteoporosis is a bone disease caused by various factors that deteriorate the microstructure of bone tissue with a decrease in BMD, lessen the mechanical strength, and increase the risk of fracture [4,26]. Calcium is one of the most important minerals that prevents osteoporosis. In postmenopausal women, a decrease in estrogen secretion accelerates bone loss and increases the risk of osteoporosis [36], and a decrease in postmenopausal BMD was observed immediately after menopause and decreased exponentially with the lapse of time, due to the continuous decrease in bone mass after menopause [37]. However, BMD only explains a portion of the bone mechanical strength variance since age-related declines in bone strength are disproportionately steeper than decreases in BMD [38]. There is no change in the biochemical composition, but, histologically, osteoporosis can be defined as a decrease in the thickness of the cortical bone and a decrease in the number and size of cancellous bone struts [3,39]. The BMC, BMD, and BV observed in the whole body, left femur, and lumbar spines (except for BMD) were increased in the OVXE group compared to the OVX group, indicating that ECO-A has a positive effect on the maintenance of bone mineralization (Figure 4, Figure 5 and Figure 6). Since BMD is not the only determinant of the bone quality [34], such as bone strength and fragility, although it is measurable value by DXA, the DXA results need to be confirmed by histological observations. It is well-recognized that rat bone is not the easiest model since specimens are small and difficult to align and match precisely for comparison [40].

In the skeleton, old bone is removed by bone resorption and new bone is produced by bone formation. Biochemical markers of this bone turnover are an index that reflects the rate of bone remodeling, and are the only noninvasive method to evaluate bone quality. While bone density is a static index of bone metabolism, biochemical markers are dynamic indicators. Deoxypyridinoline, CTXI, and cross-linked N-telopeptide of type I collagen (NTXI) are most recommended as specific bone resorption markers, whereas ALP, which is secreted by osteoblasts, and OC, are the most well-known bone formation markers [5]. This study revealed that ECO-A has positive controlling effects on these biochemical markers of bone turnover, particularly by lowering the CTXI level and maintaining the calcium concentration (Figure 7). Meanwhile, the contents of ALP and OC in serum were apparently decreased after ECO-A administration, although both are representative bone formation markers. This result can be partially explained by the previous clinical study showing that the mean level of OC was significantly raised in post-menopausal women who were osteoporotic compared to in non-osteoporotic individuals [41]. Therefore, both contents were remarkably increased in OVX groups, while they were recovered in OVXE groups to a level comparable to sham groups. Another recent study reported that the level of serum bone turnover markers, including ALP, CTXI, OC, N-terminal propeptide of type I procollagen, and tartrate-resistant acid phosphatase 5b, were all decreased in ovariectomized diabetic rats treated with salidroside [42].

A previous study revealed that when melon is treated at a high temperature (minimum 140–150 °C), the total polyphenol content and antioxidant activity become higher [43]. When the electron-donating ability was measured using the DPPH (2,2-diphenyl-1-picrylhydrazyl) assay, melon extracts showed little antioxidant activity at an untreated state, but showed a significant increase when heated. In the present study, the free radical scavenging activity of ECO-A was significantly higher than its untreated counterpart (Figure S1). It has also been reported that the ascorbic acid-equivalent antioxidant capacity of heat-treated fruit and vegetable extracts was found to increase with temperature, compared to that of untreated ones [43,44]. Particularly, the total flavonoid content and antioxidant capacity of melon after heat treatment at 150 °C were increased by more than 37 and 25 times, respectively, compared to untreated equivalents [43]. The antioxidant activity of *Citrus* peel extracts was shown to be significantly affected by the heating and processing time, suggesting that the heating process can be used as a tool for increasing their antioxidant activity [44]. This phenomenon can be partially explained by some reports showing that when fruit and vegetables were heat-treated, their antioxidant potential was increased due to the increase of free phenolic compounds, as well as the improvement of antioxidant properties of naturally occurring compounds or the formation of novel compounds, such as Maillard reaction products having antioxidant activity [45]. On the other hand, OVX rats were shown to have lower levels of antioxidant enzyme activity compared to sham rats, but the antioxidant status was significantly increased after lutein treatment [46]. Oxidative stress is known to increase the expression of cytokines in bone and thus induce osteoporosis. Antioxidants may have a role in the inhibition of the effects of free radicals on the bone and hence in the prevention of osteoporosis [47]. A recent study reported evidence to recommend the use of water as the solvent of choice for the extraction of phenolic compounds from bitter melon with antioxidant capacity [48]. Therefore, it is implicated that polyphenol-rich ECO-A possesses potent antioxidant activity and this is the reason for the protection from the decline in bone strength, mineralization, and metabolism of ovariectomized rats.

## 5. Conclusions

In conclusion, the administration of ECO-A to female rats with OVX-induced osteoporosis allowed their mechanical strengths (maximum load and stiffness) of bone to be significantly improved; their bone density of the whole body, femur, and lumbar spine to be reasonably maintained; and bone metabolism (CTXI and calcium levels) to be favorably controlled. Our findings suggest that ECO-A can be used as a protective and therapeutic agent for OVX-induced osteoporosis. In order to make sure that ECO-A can effectively protect against osteoporotic bone loss regardless of sex, it should be confirmed that it has a similar effect on male models to female ones. In the present study, however, as ECO-A was administered to ovariectomized animals, variables were not considered in the study design, such as hormonal changes in the early, middle, and late stages of osteoporosis, as well as sex. In female hypogonadal states, i.e., menopause and climacterium, it is well-recognized that bone loss accelerates with a lack of estrogen, while our knowledge of the clinical and metabolic consequences of overt male hypogonadism and the more subtle age-related decline in testosterone on bone quality is underrepresented [6]. Therefore, taking the limitations of the study design into consideration, further studies, including the mechanism of action of ECO-A and the contribution of the growth plate to bone recovery after a period of hypogonadal loss, need to be undertaken.

## Supplementary Materials

Supplementary materials can be found at https://www.mdpi.com/2076-3921/8/8/306/s1. Figure S1: Antioxidant activity of melon extracts treated without or with heat (ECO-A) determined by measuring DPPH radical scavenging activity, Table S1: Ingredient of diets used in this study.

## References

[1] Guglielmi G., Muscarella S., Bazzocchi A. (2011). Integrated imaging approach to osteoporosis: State-of-the-art review and update. Radiographics.

[2] Sözen T., Özışık L., Başaran L.Ç. (2017). An overview and management of osteoporosis. Eur. J. Rheumatol..

[3] (1993). Consensus development conference: Diagnosis, prophylaxis, and treatment of osteoporosis. Am. J. Med..

[4] NIH Consensus Development Panel (2001). Osteoporosis prevention, diagnosis, and therapy. JAMA.

[5] Dobbs M.B., Buckwalter J., Saltzman C. (1999). Osteoporosis: The increasing role of the orthopaedist. Iowa Orthop. J..

[6] Golds G., Houdek D., Arnason T. (2017). Male hypogonadism and osteoporosis: The effects, clinical consequences, and treatment of testosterone deficiency in bone health. Int. J. Endocrinol..

[7] Grodstein F., Stampfer M.J., Colditz G.A., Willett W.C., Manson J.E., Joffe M., Rosner B., Fuchs C., Hankinson S.E., Hunter D.J. (1997). Postmenopausal hormone therapy and mortality. N. Engl. J. Med..

[8] Maraka S., Kennel K.A. (2015). Bisphosphonates for the prevention and treatment of osteoporosis. BMJ.

[9] Watts N.B., Adler R.A., Bilezikian J.P., Drake M.T., Eastell R., Orwoll E.S., Finkelstein J.S. (2012). Osteoporosis in men: An Endocrine Society clinical practice guideline. J. Clin. Endocrinol. Metab..

[10] Austermann K., Baecker N., Stehle P., Heer M. (2019). Putative effects of nutritive polyphenols on bone metabolism in vivo-evidence from human studies. Nutrients.

[11] Kang S.J., Choi B.R., Kim S.H., Yi H.Y., Park H.R., Song C.H., Ku S.K., Lee Y.J. (2016). Selection of the optimal herbal compositions of red clover and pomegranate according to their protective effect against climacteric symptoms in ovariectomized mice. Nutrients.

[12] Lee S.K., Kim H., Park J., Kim H.J., Kim K.R., Son S.H., Park K.K., Chung W.Y. (2017). *Artemisia annua* extract prevents ovariectomy-induced bone loss by blocking receptor activator of nuclear factor kappa-B ligand-induced differentiation of osteoclasts. Sci. Rep..

[13] Zhang Z., Chen Y., Xiang L., Wang Z., Xiao G.G., Hu J. (2017). Effect of curcumin on the diversity of gut microbiota in ovariectomized rats. Nutrients.

[14] Jeon I.S., Kim W.D., Choi J.K. (2016). Effect of melon extracts on the cellular signaling of C2C12 osteoblast cells. Chungbuk Med. J..

[15] Ghebretinsae A.G., Thulin M., Barber J.C. (2007). Relationships of cucumbers and melons unraveled: Molecular phylogenetics of *Cucumis* and related genera (Benincaseae, Cucurbitaceae). Am. J. Bot..

[16] Garcia-Mas J., Benjak A., Sanseverino W., Bourgeois M., Mir G., González V.M., Hénaff E., Câmara F., Cozzuto L., Lowy E. (2012). The genome of melon *(Cucumis melo* L.). Proc. Natl. Acad. Sci. USA.

[17] Vouldoukis I., Lacan D., Kamate C., Coste P., Calenda A., Mazier D., Conti M., Dugas B. (2004). Antioxidant and anti-inflammatory properties of a *Cucumis melo* LC. extract rich in superoxide dismutase activity. J. Ethnopharmacol..

[18] Dhiman K., Gupta A., Sharma D.K., Gill N.S., Goyal A. (2012). A review on the medicinally important plants of the family Cucurbitaceae. Asian J. Clin. Nutr..

[19] Vella F.M., Cautela D., Laratta B. (2019). Characterization of polyphenolic compounds in cantaloupe melon by-products. Foods.

[20] Zeb A. (2016). Phenolic profile and antioxidant activity of melon (*Cucumis melo* L.) seeds from Pakistan. Foods.

[21] Heiss C., Kern S., Malhan D., Böcker W., Engelhardt M., Daghma D.E.S., Stoetzel S., Schmitt J., Ivo M., Kauschke V. (2017). A new clinically relevant t-score standard to interpret bone status in a sheep model. Med. Sci. Monit. Basic Res..

[22] Devareddy L., Hooshmand S., Collins J.K., Lucas E.A., Chai S.C., Arjmandi B.H. (2008). Blueberry prevents bone loss in ovariectomized rat model of postmenopausal osteoporosis. J. Nutr. Biochem..

[23] Kalu D.N., Salerno E., Liu C.C., Ferarro F., Arjmandi B.N., Salih M.A. (1993). Ovariectomy-induced bone loss and the hematopoietic system. Bone Miner..

[24] Sigrist I.M., Gerhardt C., Alini M., Schneider E., Egermann M. (2007). The long-term effects of ovariectomy on bone metabolism in sheep. J. Bone Miner. Metab..

[25] Qi M., Zhang L., Ma Y., Shuai Y., Li L., Luo K., Liu W., Jin Y. (2017). Autophagy maintains the function of bone marrow mesenchymal stem cells to prevent estrogen deficiency-induced osteoporosis. Theranostics.

[26] Wang F.S., Wu R.W., Lain W.S., Tsai T.C., Chen Y.S., Sun Y.C., Ke H.J., Li J.C., Hwang J., Ko J.Y. (2018). Sclerostin vaccination mitigates estrogen deficiency induction of bone mass loss and microstructure deterioration. Bone.

[27] Abe T., Chow J., Lean J.M., Chambers T.J. (1993). Estrogen does not restore bone lost after ovariectomy in the rat. J. Bone Miner. Res..

[28] Noh D., Lim Y., Lee H., Kim H., Kwon O. (2018). Soybean-hop alleviates estrogen deficiency-related bone loss and metabolic dysfunction in ovariectomized rats fed a high-fat diet. Molecules.

[29] Kim G.H., Baek H.K., Lee J.S., Kim S.J., Yi S.S. (2019). Chronic oral administration of *Tenebrio molitor* extract exhibits inhibitory effect on glucocorticoid receptor overexpression in the hippocampus of ovariectomy-induced estrogen deficient mice. J. Food Sci..

[30] Shahnazari M., Martin B.R., Legette L.L., Lachcik P.J., Welch J., Weaver C.M. (2009). Diet calcium level but not calcium supplement particle size affects bone density and mechanical properties in ovariectomized rats. J. Nutr..

[31] Van Pelt R.E., Gavin K.M., Kohrt W.M. (2015). Regulation of body composition and bioenergetics by estrogens. Endocrinol. Metab. Clin. North Am..

[32] Fan J.Z., Wang Y., Meng Y., Li G.W., Chang S.X., Nian H., Liang Y.J. (2015). *Panax notoginseng* saponins mitigate ovariectomy-induced bone loss and inhibit marrow adiposity in rats. Menopause.

[33] Fonseca H., Moreira-Gonçalves D., Coriolano H.J., Duarte J.A. (2014). Bone quality: The determinants of bone strength and fragility. Sports Med..

[34] Tanaka S.M., Yorozuya Y., Takatsu D. (2017). Random electromyostimulation promotes osteogenesis and the mechanical properties of rat bones. Ann. Biomed. Eng..

[35] Shaban N.Z., Talaat I.M., Elrashidy F.H., Hegazy A.Y., Sultan A.S. (2017). Therapeutic role of *Punica granatum* (pomegranate) seed oil extract on bone turnover and resorption induced in ovariectomized rats. J. Nutr. Health Aging.

[36] Levin V.A., Jiang X., Kagan R. (2018). Estrogen therapy for osteoporosis in the modern era. Osteoporos. Int..

[37] Fakkert I.E., Teixeira N., Abma E.M., Slart R., Mourits M., de Bock G.H. (2017). Bone mineral density and fractures after surgical menopause: Systematic review and meta-analysis. BJOG.

[38] Keaveny T.M., Kopperdahl D.L., Melton L.J., Hoffmann P.F., Amin S., Riggs B.L., Khosla S. (2010). Age-dependence of femoral strength in white women and men. J. Bone Miner. Res..

[39] Kanis J.A., McCloskey E.V., Harvey N.C., Johansson H., Leslie W.D. (2015). Thresholds and the diagnosis of osteoporosis. J. Bone Miner. Res..

[40] Bell S., Ajami E., Davies J.E. (2014). An improved mechanical testing method to assess bone-implant anchorage. J. Vis. Exp..

[41] Kalaiselvi V.S., Prabhu K., Ramesh M., Venkatesan V. (2013). The association of serum osteocalcin with the bone mineral density in post menopausal women. J. Clin. Diagn. Res..

[42] Zheng H., Qi S., Chen C. (2018). Salidroside improves bone histomorphology and prevents bone loss in ovariectomized diabetic rats by upregulating the OPG/RANKL ratio. Molecules.

[43] Zou Z., Xi W., Hu Y., Nie C., Zhou Z. (2016). Antioxidant activity of *Citrus* fruits. Food Chem.

[44] Kim H.Y., Woo K.S., Hwang I.G., Lee Y.R., Jeong H.S. (2008). Effects of heat treatments on the antioxidant activities of fruits and vegetables. Korean J. Food Sci. Technol..

[45] Turkmen N., Sari F., Velioglu Y.S. (2005). The effect of cooking methods on total phenolics and antioxidant activity of selected green vegetables. Food Chem..

[46] Li H., Huang C., Zhu J., Gao K., Fang J., Li H. (2018). Lutein suppresses oxidative stress and inflammation by Nrf2 activation in an osteoporosis rat model. Med. Sci. Monit..

[47] Abdollahi M., Larijani B., Rahimi R., Salari P. (2005). Role of oxidative stress in osteoporosis. Therapy.

[48] Tan S.P., Stathopoulos C., Parks S., Roach P. (2014). An Optimised aqueous extract of phenolic compounds from bitter melon with high antioxidant capacity. Antioxidants.

