# Supplementary material for: Protective Effects of Melon Extracts on Bone Strength, Mineralization, and Metabolism in Rats with Ovariectomy-Induced Osteoporosis
Source: Antioxidants (Basel). 2019 Aug 14;8(8):306. doi: 10.3390/antiox8080306 (PMC6720457; doi:10.3390/antiox8080306)
Supplement: Supplementary file 1 [file antioxidants-08-00306-s001.pdf]

Supplementary Materials: Protective Effects of Melon Extracts on Bone Strength, Mineralization, and Metabolism in Rats with Ovariectomy-induced Osteoporosis

Bongju Kim, Sung-Ho Lee, Su-Jin Song, Won Hyeon Kim, Eun-Sung Song, Jae-Chang Lee, Sung-Jae Lee, Dong-Wook Han, Jong-Ho Lee

Table S1. Ingredient of diets used in this study.

**Ingredient (g/kg Diet)**	
Sucrose	500
Casein	200
Corn starch	150
Cellulose	50
Soybean oil	50
Mineral mixture	35
Vitamin mixture	10
L-Cystein	3
Choline bitartrate	2
Carbohydrate (% kcal)	64
Protein (% kcal)	20
Fat (% kcal)	16
Energy (kcal/g)	4

DPPH Assay

The antioxidant activity of melon extracts treated without or with heat (ECO-A) was measured by the DPPH (2,2-diphenyl-1-picrylhydrazyl) assay as described elsewhere [1]. The discoloration of the reaction mixture was measured at 520 nm for 30 min of incubation in the dark. Ascorbic acid solution in DW was used as a reference standard and DW was used as blank (control). The antioxidant activity (%) was defined as the percentage scavenging of the DPPH free radical from the following equation:

$$\text{Percentage scavenging} = A_0 - A_1/A_1 \times 100$$

where A_0 = Absorbance of the control and A_1 = Absorbance in the presence of the sample.

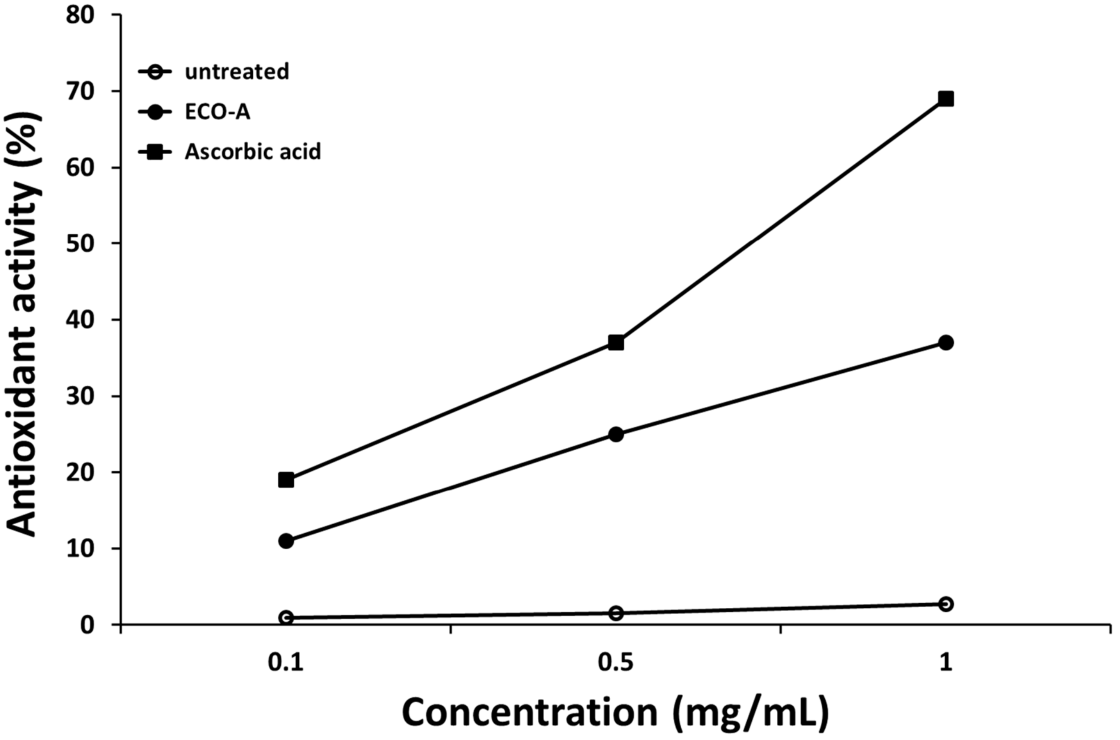

Figure S1. Antioxidant activity of melon extracts treated without or with heat (ECO-A) determined by measuring DPPH radical scavenging activity.

Reference

1. Usha, T.; Middha, S.K.; Bhattacharya, M.; Lokesh, P.; Goyal, A.K. Rosmarinic acid, a new polyphenol from *Baccaurea ramiflora* Lour. leaf: A probable compound for its anti-inflammatory activity. *Antioxidants* **2014**, *3*, 830–842.
